# A novel fluorescent probe with a phosphofluorene molecular structure for selective detection of hydrogen sulfide in living cells†

**DOI:** 10.1039/d4ra02979h

**Published:** 2024-07-02

**Authors:** Shuntao Zhang, Xingyong Liu, Xiangjun Chen, Jiefeng Tang, Juan Wang

**Affiliations:** a College of Chemical Engineering, Sichuan University of Science & Engineering Zigong 643000 China wangjuan2022@suse.edu.cn

## Abstract

Hydrogen sulfide (H_2_S) gas plays a significant role in biological regulation. With advancements in technology, H_2_S has been discovered across diverse fields, necessitating a comprehensive understanding of its physiological functions through monitoring changes in H_2_S within complex environments and physiological processes. In this study, we designed a phosphofluorene-based conjugate probe PPF-CDNB with an asymmetric π-conjugated phosphine structure and utilized dinitrophenyl ether as the recognition site for H_2_S. PPF-CDNB exhibited exceptional resistance to interference and demonstrated stability over a broad pH range (3.0–10.0), making it suitable for various environmental conditions. Intracellular experiments revealed that PPF-CDNB effectively monitored both endogenous and exogenous levels of H_2_S.

## Abstract 2

The fluorescent probe PPF-CDNB based on the asymmetric π-conjugated phosphine structure of phosphorfluorene can highly selectively and sensitively detect hydrogen sulfide in live cells, and exhibits a significant fluorescence enhancement effect.

## Graphical abstract

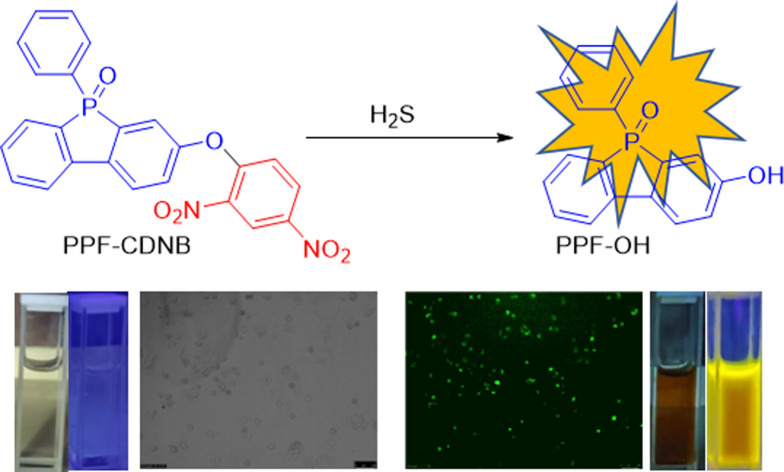

## 1 Introduction

Hydrogen sulfide (H_2_S) is a gaseous compound characterized by an unpleasant odor reminiscent of rotten eggs. It is frequently encountered in heavy industrial processes, such as metal smelting and coal mining, as well as during the prolonged decay and fermentation of organic matter.^1–4^ H_2_S can induce sensory discomfort, stimulate the nervous system, and in severe cases lead to poisoning or even rapid death.^5–7^ Functioning as a signaling molecule, H_2_S plays a pivotal role in several crucial physiological processes including blood pressure regulation, nerve conduction, and oxidative stress inhibition.^8–10^ Elevated levels of H_2_S have been associated with various conditions including Alzheimer's disease, Down syndrome, Parkinson's disease, and diabetes.^11,12^ Hydrogen sulfide represents a bioactive molecule that assumes critical regulatory functions within biological systems.^13,14^ However, aberrant levels of H_2_S are closely associated with functional impairments and various diseases, prompting active development of small molecule chemical tools for investigating its diverse roles in biology and medicine.^15^ In addition to its lethal effects, H_2_S also modulates a wide range of physiological actions including vasodilation, anti-inflammatory effects, insulin release, neurotransmission, antioxidant properties, anti-apoptotic effects, and neuroprotection.^16^ Notably, H_2_S triggers *S*-sulfhydration of Keap1, activating Nrf_2_ and facilitating its nuclear translocation, which results in the production of antioxidant proteins.^17^ Numerous studies have demonstrated that H_2_S ranks as the third most crucial signaling molecule in organisms following CO and NO.^18,19^ Conventional methods for H_2_S detection, including electrochemical analysis, methylene blue assay, colorimetric assay, spectrophotometry, and chromatography, are often time-consuming and labor-intensive, limiting their effectiveness in providing accurate and efficient qualitative and quantitative analysis of H_2_S in cells.^20–22^ Thus, there is a pressing need for simple and rapid detection methods to measure H_2_S concentration accurately in various environments, including water phase, gas phase, and living cells (both exogenous and endogenous). Among the available detection methods, the fluorescence probe detection method has gained significant attention due to its high sensitivity, selectivity, cost-effectiveness, ease of operation, and real-time determination.

In our study, we utilized the phosphofluorene (PPF) structure as the probe fluorophores.^23–26^ PPF, also known as dibenzo phosphocyclopentadiene, features a robust ring structure composed of two benzene rings and a phosphocyclopentadiene core.^27–31^ Similar to fluorene, PPF exhibits a wide energy gap, and its fluorescence spectrum is predominantly observed in the ultraviolet region (366 nm).^32,33^ The presence of two lateral benzene rings in PPF provides the system with several advantages over monocyclic phosphoheterocycles in binding molecular electronics.^34,35^ The synthesis and operation of the tricyclic system can modulate the overall electronic structure through the introduction and manipulation of benzene ring substituents.^36,37^ PPF exhibits excellent fluorescence and its rigid configuration ensures a high photoluminescence quantum yield.^38–41^ Despite these advantages, the use of PPF structures in fluorescent probes is relatively uncommon. Most commercially available H_2_S fluorescent probes rely on reduction and nucleophilic reactions for detection, which suffer from slow reaction rates leading to inefficient detection processes lasting tens of minutes or even hours. For instance, Kim's group designed the fluorescent probe BT-ITC, which showed emission at 447 nm and had a response time of 40 min to H_2_S.^42^ Fang's group designed the fluorescent probe Mito-GW, which has a response time of 80 min to H_2_S and displays emission at 447 nm.^43^ Zhong's group designed a fluorescent probe, THQ-L, that displayed emission at 650 nm with a response time of 45 min to H_2_S.^44^ While these results contribute to the advancement of fluorescent probes, they also have limitations, notably long response times (exceeding 20 minutes). Therefore, there is a critical need to design a fluorescent probe with rapid response, high selectivity, and sensitivity to H_2_S detection. In response to this need, we designed and synthesized a fluorescent probe, PPF-CDNB. The phosphomfluorene derivative of the probe was used as a fluorophore, while 2,4-dinitrophenyl was employed as a functional recognition group. PPF-CDNB exhibits a wide pH range (3.0–10.0), excellent sensitivity (LOD = 150 nM), and high selectivity for H_2_S detection, minimizing potential interference. Thus, PPF-CDNB offers a fast and efficient method for detecting H_2_S.

## 2 Experimental

### 2.1 Instruments and reagents

All reagents and solvents were purchased from commercial suppliers and used without further purification. Distilled water was utilized in the experiment after passing through a water superpurification system. Silica column chromatography was conducted using 200–300 mesh silica and an appropriate solvent. The reaction progress was monitored by thin-layer chromatography (TLC), and the reaction components were visualized using UV light. Fluorescence spectra and relative fluorescence intensities were measured using the Shimadzu RF-5301 fluorescence spectrometer. The excitation wavelength for all fluorescence measurements was 294 nm, with excitation and emission slit widths set to 2.5 nm. Ultraviolet-visible spectroscopy was performed using the Shimadzu UV-2700 spectrophotometer. The ^1^H-NMR and ^13^C-NMR spectra were recorded on the BRUKER600 spectrometer. pH measurements were conducted using a PHS-3C pH meter. Electrospray mass spectrometry (ESI-MS) data were acquired using the Agilent 1100 series instrument. Cell images were captured using a fluorescence microscope (Leica, Germany).

### 2.2 Synthesis of probe PPF-CDNB

**Fig. 1:**
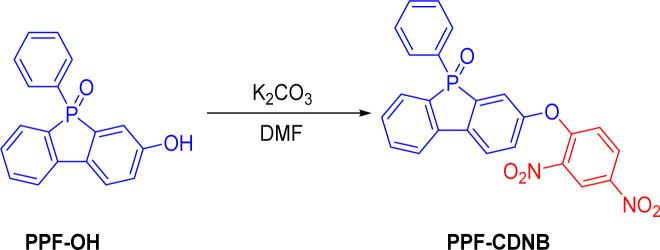
Synthesis of probe PPF-CDNB.

PPF-OH (200 mg, 0.68 mmol) and 2,4-dinitrophenyl chloride (166.4 mg, 0.82 mmol) were dissolved in 7 mL DMF and added to a round-bottom flask. Potassium carbonate (141 mg, 1.03 mmol) was then added, and stirred at 100 °C for 6 h. After the reaction, the mixture was extracted with ethyl acetate, and the ethyl acetate extract was passed through a column. The column was eluted with a mixture of ethyl acetate and petroleum ether to yield a yellow solid compound, PPF-CDNB (267 mg, 85.6%) (Fig. 1). ^1^H-NMR (600 MHz, CDCl_3_) *δ* 8.78 (d, *J* = 2.7 Hz, 1H), 8.28 (dd, *J* = 9.2, 2.7 Hz, 1H), 7.86 (dd, *J* = 8.4, 3.2 Hz, 1H), 7.78 (dd, *J* = 7.7, 2.8 Hz, 1H), 7.68 (dd, *J* = 9.8, 7.6 Hz, 1H), 7.57 (dt, *J* = 8.2,4.4 Hz, 4H), 7.47 (td, *J* = 7.4, 1.2 Hz, 1H), 7.41–7.34 (m, 5H), 7.29 (dd, *J* = 8.4, 2.2 Hz, 1H), 7.06 (d, *J* = 9.2 Hz, 1H). ^13^C-NMR (150 MHz, CDCl_3_) *δ* 154.04, 153.69, 153.59, 141.05, 139.68, 139.55, 138.81, 138.67, 132.88, 131.74, 130.04, 129.96, 129.28, 129.22, 128.85, 128.78, 128.05, 128.03, 127.94, 124.30, 122.44 (d, *J* = 11.5 Hz), 121.20, 120.36 (d, *J* = 10.0 Hz). HRMS (ESI): C_24_H_15_N_2_O_6_NaP for [M + Na]^+^, calculated 481.0565, found 481.0570.

### 2.3 Fluorometric measurements

To further investigate the performance of the probe, spectral experiments were conducted. Initially, a detection solution was prepared by weighing an appropriate amount of probe PPF-CDNB and dissolving it in DMF solution to create a 1 mM stock solution. Subsequently, 100 μL of the probe stock solution was added to a volumetric flask containing PBS/DMF (v/v = 9 : 1, pH = 7.4) buffer solution to achieve a concentration of 20 μM. Spectral measurements were then conducted in both the presence and absence of analytes Typically, the reaction between probe PPF-CDNB and NaHS occurs within the PBS/DMF (v/v = 9 : 1, pH = 7.4) system followed by measuring the fluorescence intensity at 528 nm in the reaction solution.

### 2.4 Selectivity and specificity

The probe stock solution (100 μL) was prepared in DMF. Various test substances (K^+^, Cu^+^, Ca^2+^, Na^+^, I^−^, F^−^, Cl^−^, CO_3_^2−^, HCO_3_^−^, S_2_O_8_^2−^, HSO_4_^−^, HSO_3_^−^, SCN^−^, S_2_O_3_^2−^, S_2_O_5_^2−^, SO_3_^2−^, NO_2_^−^, l-Cys, d-Cys, GSH, Zn^2+^) were prepared in distilled water. All anions are prepared from their sodium salts, and all cations are prepared from their chloride salts. The resulting solution was stored at room temperature (25 °C), and then the fluorescence spectrum was recorded.

### 2.5 Cell cultures and imaging

For exogenous imaging, A549 cells were incubated with PPF-CDNB (10 μM) at 37 °C for 30 minutes and then cultured with NaHS (50 μM) for 30 minutes. For endogenous imaging, the A549 cells were divided into three plates. One plate was incubated with cysteine (Cys, 50 μM) and PPF-CDNB (10 μM) for 30 minutes. The second plate was incubated with dl-propyl glycine (PAG, 100 μM) and PPF-CDNB (10 μM) for 30 min, and the third plate was incubated with dl-propyl glycine (PPG, 100 μM), cysteine (Cys, 50 μM) and PPF-CDNB (10 μM) for 30 min. The images were obtained under a fluorescence microscope.

## 3 Discussion and experimental

### 3.1 Design idea

PPF and its derivatives have demonstrated strong fluorescence properties.^45,46^ This probe introduces 2,4-dinitrophenyl ether with dual functions: (a) inhibition of the intramolecular charge transfer (ICT) process of the fluorophores of PPF derivatives; (b) serving as a recognition site for H_2_S. By combining the ICT of the PPF derivative with the signal reporter mechanism of photoinduced electron transfer, we designed and synthesized a diaryl ether struct-based probe PPF-CDNB. The PPF-CDNB probe utilizes phosphofluorenine as the luminescence group, connected by a simple and rigid π-conjugated structure to the hydroxyl group of the electron donor group. It exhibits typical ICT luminescence characteristics, excellent photostability and high fluorescence quantum yield. The 2,4-dinitrophenyl ether group in the probe molecule serves as a potent electron-absorbing moiety, capable of quenching the fluorescence of the luminescent group *via* a photoinduced electron transfer process. The dinitrophenyl ether functions as the recognition site for H_2_S. Upon encountering H_2_S, a thiolysis reaction occurs, blocking the PET process and leading to the recovery of fluorescence from the fluorophore. This results in a reactive H_2_S fluorescent probe with significantly enhanced fluorescence.

### 3.2 Photophysical properties of the PPF-CDNB probe

**Fig. 2:**
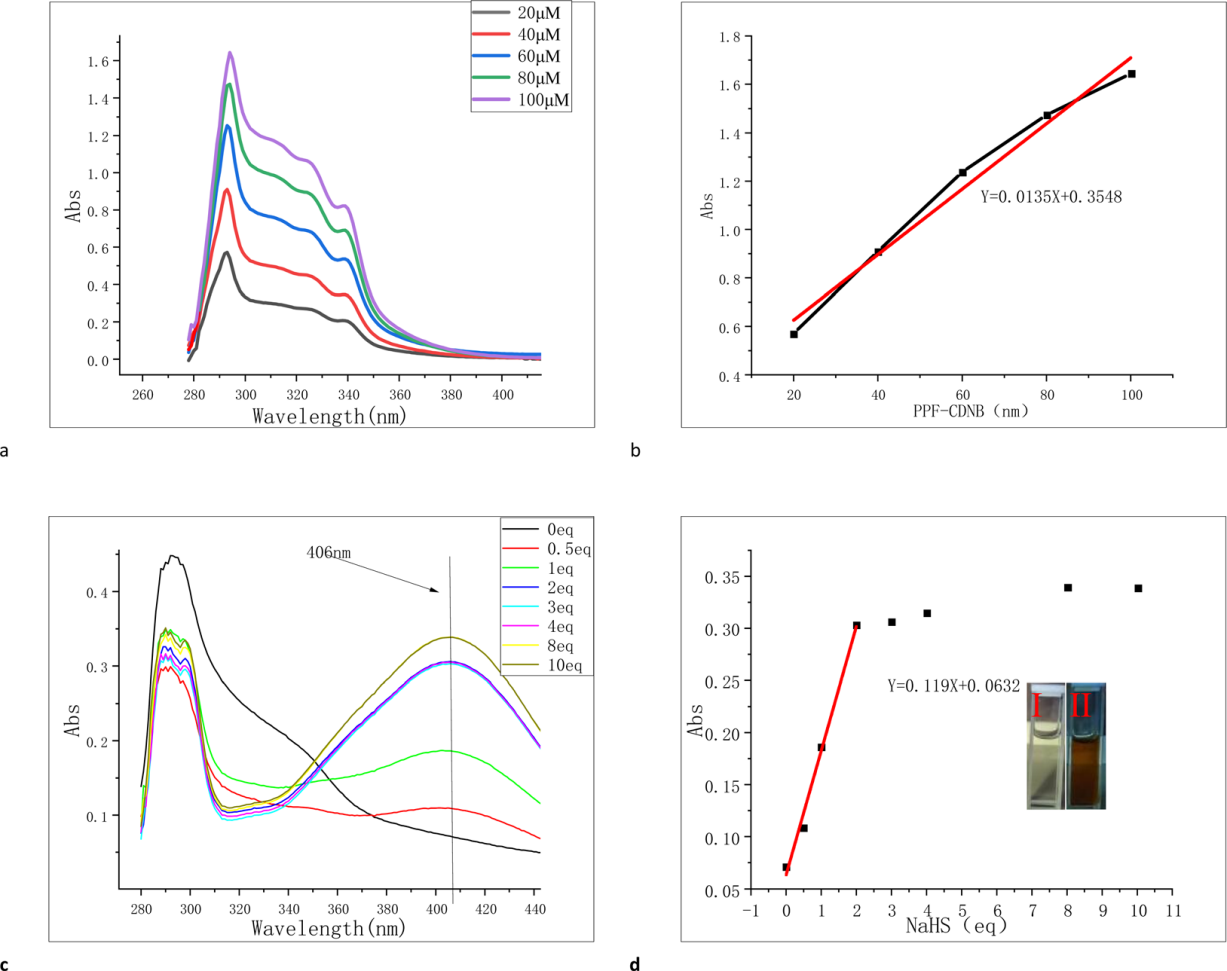
(a) UV-vis spectra of PPF-CDNB fluorescent probes at different concentrations (20–100 μM). (b) Linear dependence of the UV absorbance at 293 nm with an equal gradient of concentration for different concentrations of PPF-CDNB fluorescent probes. (c) UV absorption spectra of 20 μM probe PPF-CDNB at 15 min after the addition of NaHS concentration (0–10 eq.). (d) Linear relationship between probe PPF-CDNB (20 μM) at 406 nm and NaHS concentration (0–10 eq.). In PBS/DMF (V/V = 9/1, pH 7.4) buffer solution system.

For probe PPF-CDNB, 10% DMF was added to PBS buffer solution to enhance its solubility and fluorescence (Fig. S1†). We investigated the interaction of probe PPF-CDNB (20 μM) with H_2_S in a PBS/DMF (V/V = 9/1, pH 7.4) system by UV absorption spectroscopy. As shown in the Fig. 2, the maximum UV absorption of PPF-CDNB probe is located at 294 nm. By increasing the concentration of PPF-CDNB probe (20–100 μM) (Fig. 2a), the absorbance coefficient at 294 nm increases linearly, according to the linear regression equation (*R*^2^ = 0.975) (Fig. 2b). When the PPF-CDNB probe concentration was 20 μM, the absorbance at 294 nm was obviously decreased and the UV absorbance at 406 nm was significantly increased by adding NaHS solution (0–10 eq.) (Fig. 2c). The color of the solution changed from colorless (Fig. 2d(I)) to brown-yellow (Fig. 2d(II)), and new compounds were formed.

### 3.3 Effect of pH on H_2_S recognition by the PPF-CDNB probe

**Fig. 3:**
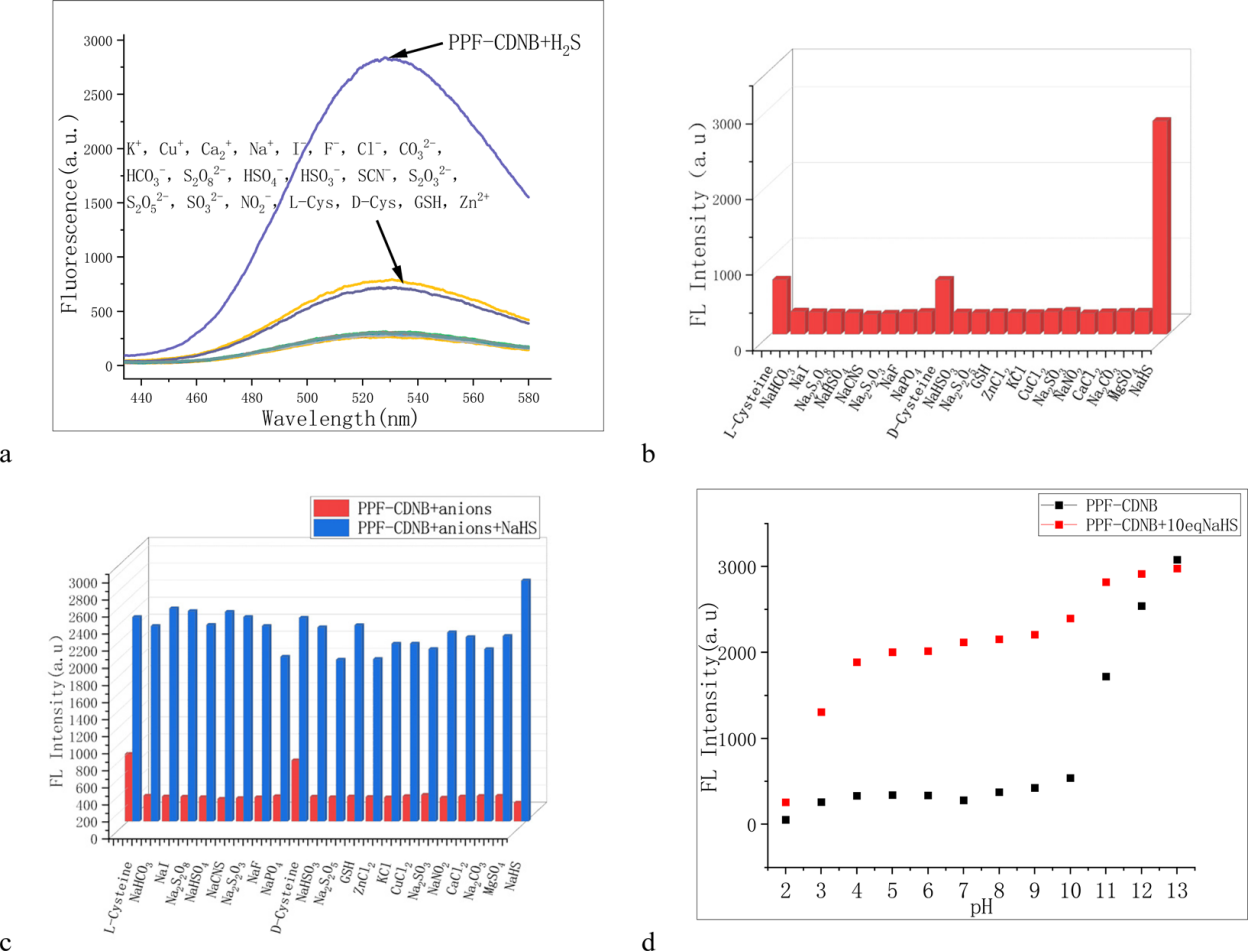
(a) Fluorescence spectra of probe PPF-CDNB (20.0 μM) after adding 22 analytes of 200 μM. (b) Bar chart showing the selectivity of other analytes to probe H_2_S detection. (c) Bar chart showing the anti-interference of other analytes to probe H_2_S detection. (d) Fluorescence intensity of 20 μM probe PPF-CDNB at different pH values the pH was adjusted with NaOH and HCl, and the fluorescence intensity at 528 nm at different pH values. In a buffer solution system of PBS/DMF (V/V = 9/1, pH 7.4).

For successful bioluminescence imaging, the PPF-CDNB probe must be activated within the appropriate physiological pH range to investigate the effects of different pH conditions on its performance (Fig. 3d). It was observed that the PPF-CDNB probe was basically unaffected by pH in the pH range of 4.0–10.0. However, upon addition of H_2_S, the fluorescence intensity of the PPF-CDNB probe at 528 nm significantly increased. Within the pH range of 1.0–4.0, as the acidity increases, the fluorescence intensity of both PPF-CDNB probe and PPF-CDNB probe with H_2_S significantly decreases (Fig. S2†). It is speculated that the structure of the PPF of the PPF-CDNB probe is disrupted, which disrupts the ESIPT process in the PPF fluorophore. In contrast, within the pH range of 10.0–13.0, the fluorescence intensity of both the PPF-CDNB probe and the PPF-CDNB probe with H_2_S increased significantly with increasing alkalinity (Fig. S2†). It is speculated that disruption in the structure of the 2,4-dinitrophenyl ether moiety blocked the PET process, leading to restoration of fluorophore fluorescence. These findings indicate that the probe's response to H_2_S spans across acidic and alkaline conditions, enabling its applicability in various environments such as water phase, gas phase, and live cell measurements. This broad response range enhances its utility and value.

### 3.4 The selectivity and competitiveness of PPF-CDNB probe for H_2_S

The detection system often comprises a variety of chemical substances, potentially interfering with the identification of target products. Therefore, the selectivity of PPF-CDNB probe with various ions was studied in PBS/DMF (V/V = 9/1, pH 7.4) system. In the dispersed system of PPF-CDNB (20 μM) buffer solution, 200 μM of K^+^, Cu^+^, Ca^2+^, Na^+^, I^−^, F^−^, Cl^−^, CO_3_^2−^, HCO_3_^−^, S_2_O_8_^2−^, HSO_4_^−^, HSO_3_^−^, SCN^−^, S_2_O_3_^2−^, S_2_O_5_^2−^, SO_3_^2−^, NO_2_^−^, l-Cys, d-Cys, GSH, and Zn^2+^ were separately added, and the fluorescence spectra at 528 nm were measured after 10 minutes of incubation. Fluorescence spectra at 528 nm after 10 min of action (Fig. 3a and b). The addition of organic salt and inorganic salt had no significant effect on the fluorescence intensity of PPF-CDNB probe. Although the addition of l-Cys and d-Cys can enhance the fluorescence intensity of PPF-CDNB probe system, it was significantly lower than that caused by the addition of NaHS (Fig. 3c). Notably, when interfering ions were added to the probe, the fluorescence intensity was greatly enhanced upon the addition of NaHS, reaching a level comparable to the fluorescence intensity without interfering ions. Therefore, the PPF-CDNB probe exhibits good selectivity for H_2_S detection.^47^ The detection limit (LOD) of the fluorescence probe was calculated to be 0.15 μM (LOD = 3*σ*/*k*) after analysis (Fig. S3†). The PPF-CDNB probe can accurately recognize H_2_S even under very harsh conditions.

### 3.5 Calculation of DFT

**Fig. 4:**
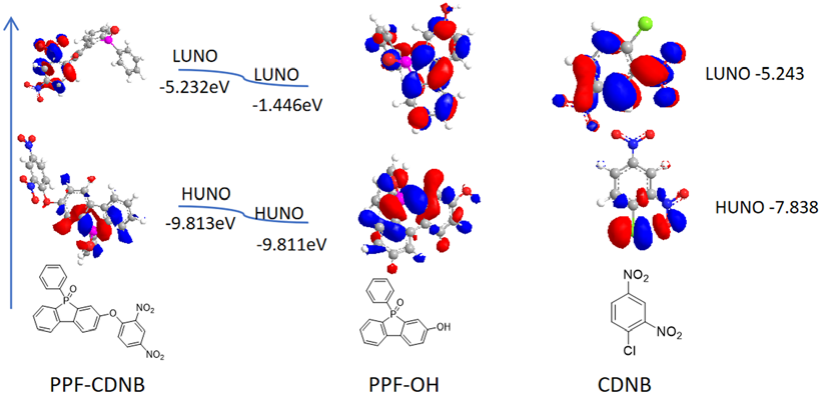
Structure optimization of compounds PPF-CDNB, PPF-OH and CDNB by DFT.

To comprehensively understand the fluorescence change mechanism of probes PPF-CDNB and PPF-OH, DFT calculations were performed using the B3LYP/6-311g(d) level in the Gauss 09 program. A polarizable continuum model (PCM) was employed to account for solvent effects in DMF. The LUMO energy of 1-chloro-2,4-dinitrobenzene (−5.243 eV) falls between the HOMO energy of compound PPF-OH (−9.811 eV) and its LUMO energy (−1.446 eV), indicating that PET can occur (Fig. 4).

### 3.6 Study on the kinetics of H_2_S by PPF-CDNB probe

**Fig. 5:**
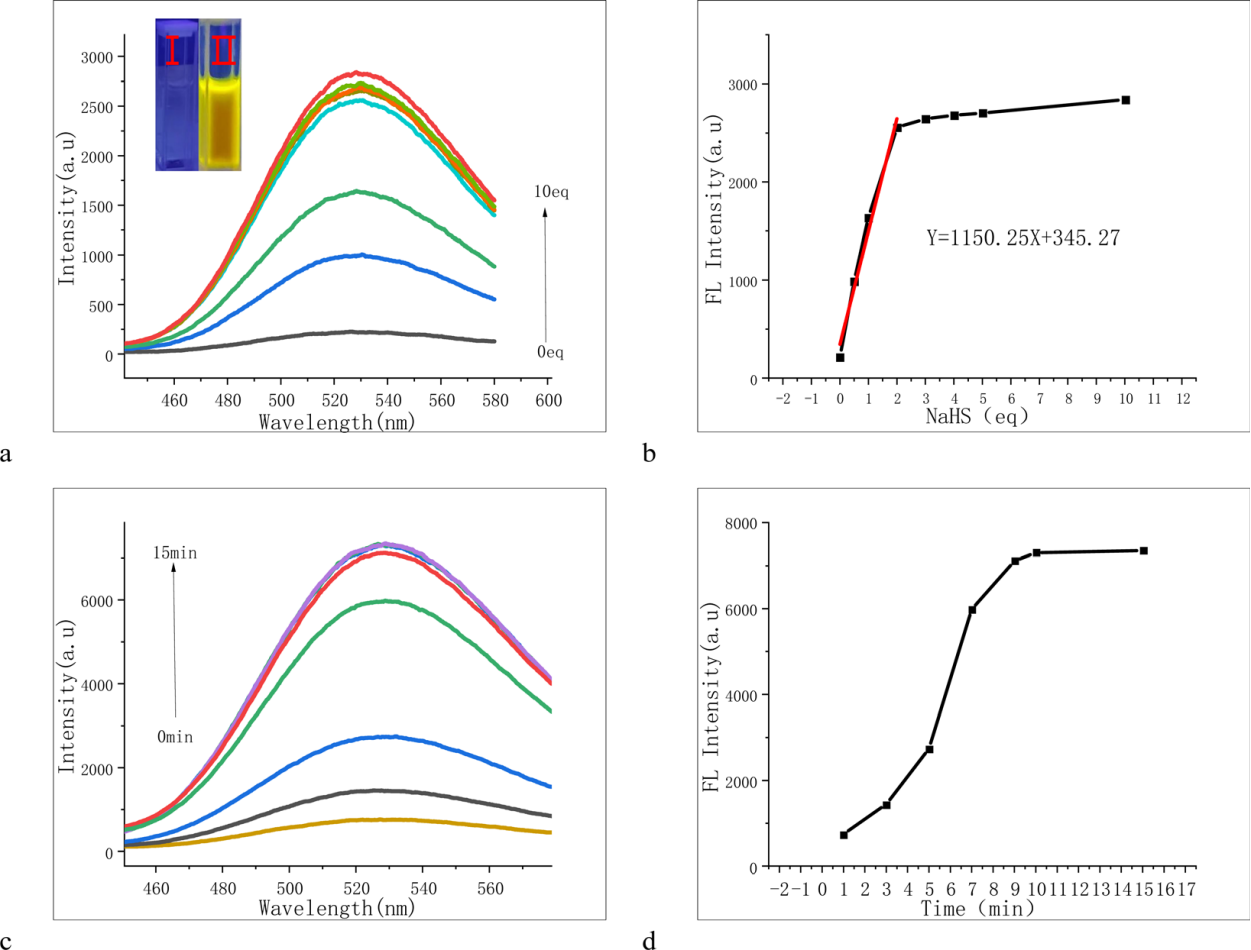
Fluorescence titration curve of probe PPF-CDNB was obtained. (a) The fluorescence emission spectrum of 20 μM probe PPF-CDNB was recorded at 15 minutes after the addition of NaHS concentration (0–10 eq.). (b) A linear relationship between the fluorescence intensity of probe PPF-CDNB (20 μM) at 528 nm and NaHS concentration (0–2 eq.) was observed. (c) The fluorescence emission spectra of the 20 μM probe PPF-CDNB were measured at 10 eq. NaHS for 0–15 minutes. (d) Curve of fluorescence intensity of probe PPF-CDNB (20 μM) *versus* the time gradient between 0 and 15 min in the presence of 10 eq. NaHS in PBS/DMF buffer (V/V = 9/1, pH 7.4) at 528 nm with an excitation wavelength of 293 nm.

UV-visible and fluorescence titration experiments were conducted in PBS/DMF (V/V = 9/1, pH 7.4) buffer solution. As depicted in the figures, the fluorescence intensity at 528 nm gradually increased with the addition of NaHS, becoming notably enhanced at 2 equivalents (Fig. 5a and b). Concurrently, the solution transitioned from colorless to yellow, emitting strong yellow fluorescence under a 365 nm UV lamp (Fig. 5(I, II) and S5†). We also observed a time-dependent phenomenon with the PPF-CDNB probe (Fig. 5c and d). In the PBS/DMF system (V/V = 9/1, pH = 7.4), at a concentration of 20 μM, the fluorescence intensity of PPF-CDNB gradually rose within the first 10 minutes, reaching its peak at 10 minutes, and then stabilized. This experiment demonstrated that the fluorescence intensity of PPF-CDNB exhibited a linear relationship with the concentration of NaHS in the range of 0–2 equivalents, confirming the utility of PPF-CDNB as a tool for H_2_S detection (Fig. 5b).

### 3.7 Sensing mechanism

**Fig. 6:**
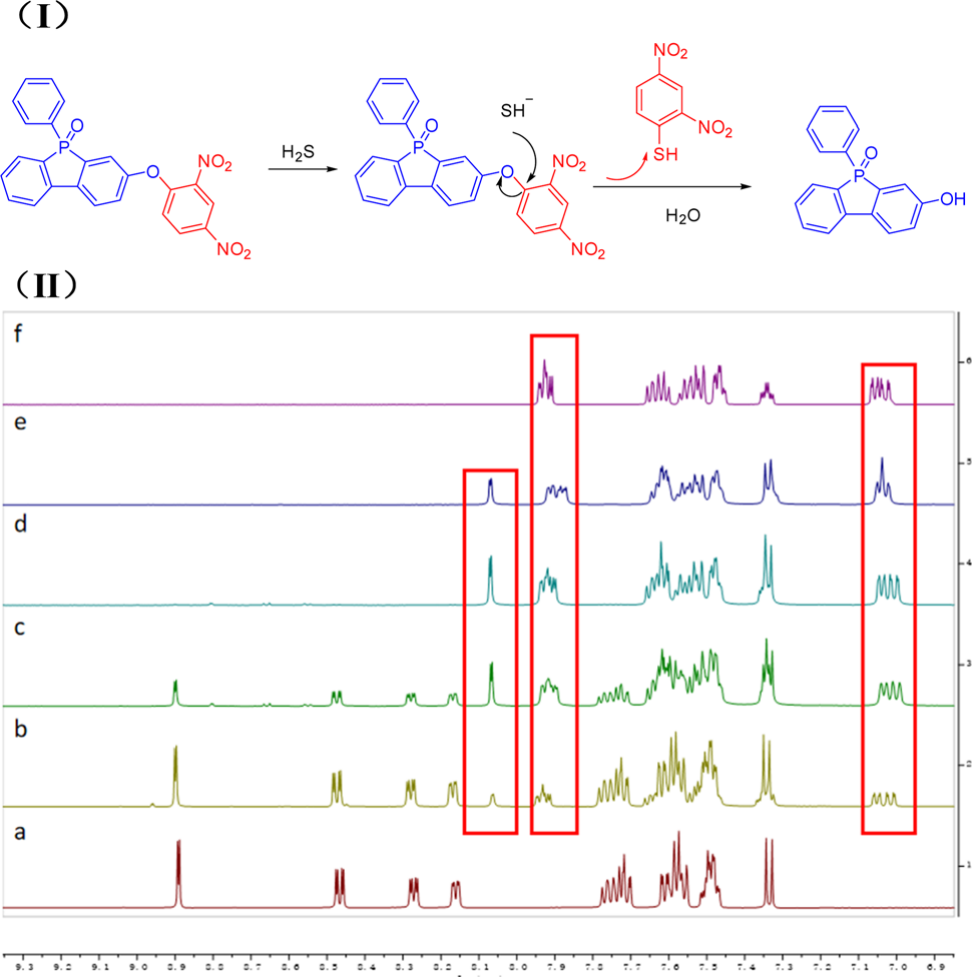
(I) Possible mechanism for the probe PPF-CDNB with H_2_S. (II) ^1^H-NMR titration experiments of probe PPF-CDNB. (a) PPF-CDNB. (b) PPF-CDNB + 1 eq. NaHS. (c) PPF-CDNB + 3 eq. NaHS. (d) PPF-CDNB + 5 eq. NaHS. (e) PPF-CDNB + 10 eq. NaHS. (f) PPF-OH. ^1^H-NMR measurements were performed 10 min after addition of NaHS to PPF-CDNB (600 MHz, DMSO-d6).

To further elucidate the reaction mechanism between PPF-CDNB and H_2_S, we conducted a ^1^H-NMR titration experiment. Confirmation of the conversion of PPF-CDNB to PPF-OH was achieved through ^1^H-NMR titration of PPF-CDNB in the presence of H_2_S in a DMSO-d6 solution (Fig. 6(II)). It was observed that the peak of 2,4-dinitrobenzene on PPF-CDNB gradually decreased with the increase of NaHS after the addition of 1 eq., 3 eq., 5 eq., and 10 eq. NaHS, respectively. Following the addition of 5 eq. NaHS, the bimodal proton signal at 8.89 ppm, 8.47 ppm, 8.27 ppm, and 8.16 ppm disappeared. It moved to the high-field region again, appearing at 8.07 ppm, 7.60–7.62 ppm and 7.33 ppm. Upon adding 10 equivalents of NaHS, the peak position remained essentially unchanged compared to adding 5 equivalents of NaHS, indicating that excess H_2_S did not affect the thiolysis reaction of PPF-CDNB. These findings support a sensing mechanism involving hydrogen sulfide-induced ether bond thiolysis (Fig. 6(I)). Additionally, the sensing mechanism was corroborated by HPLC analysis (Fig. S6†).

### 3.8 Live cell fluorescence imaging

Inspired by the above experimental results, we further investigated the practicality of probes for detecting intracellular H_2_S. Initially, we conducted standard studies to evaluate the cytotoxicity of the probes. In preliminary experiments, the cytotoxicity of PPF-CDNB on A549 cells was examined using a CCK-8 assay (Fig. S7†). Our observations revealed that when treated with 10 μM PPF-CDNB for 12 hours, approximately 80% of A549 cells remained viable. The results indicate that the probe exhibited low toxicity to A549 cells (Fig. S7†).

**Fig. 7:**
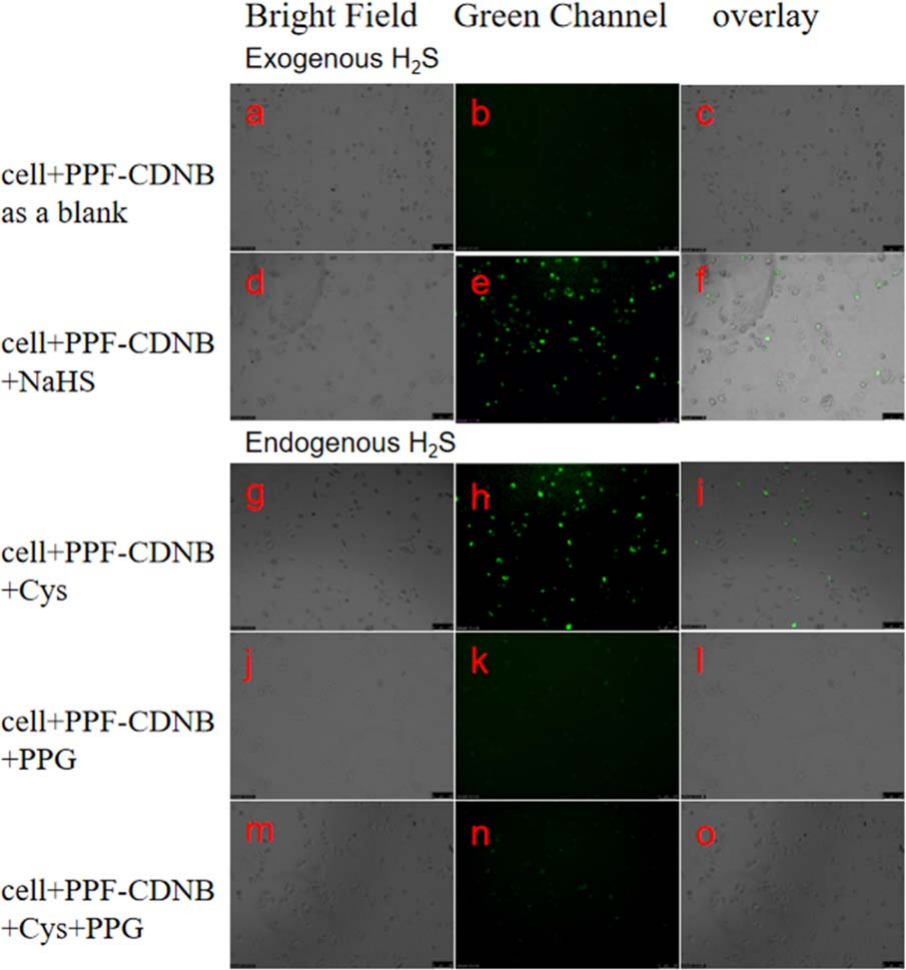
Images of A549 cells co-cultured with 10 μM PPF-CDNB for 30 minutes. (a) Bright field. (b) Green channel. (c) Overlay. (d–f) Bright field, green channel, and overlay images of A549 cells treated with 50 μM NaHS. (g–i) Bright field, green channel, and overlay images of A549 cells treated with 50 μM Cys. (j–l) Bright field, fluorescence images, and overlay of A549 cells treated with 200 μM PPG. (m–o) Bright field, green channel, and overlay images of A549 cells treated with 200 μM PPG followed by 50 μM Cys.

We utilized PPF-CDNB to detect both exogenous and endogenous H_2_S in living A549 cells, selecting a concentration of 10 μM based on cytotoxicity assays. Exogenous H_2_S detection was divided into two groups. In the first group, A549 cells were pre-incubated with PPF-CDNB for 30 minutes as a blank control, resulting in minimal fluorescence under fluorescence microscopy (Fig. 7b). In the second group, A549 cells were pre-incubated with PPF-CDNB followed by 50 μM NaHS, leading to pronounced green fluorescence in the cytoplasmic region of the cells (Fig. 7e). Demonstrate the decomposition of PPF-CDNB by exogenous H_2_S into PPF-OH, which emits dazzling fluorescence. Light field images of A549 cells incubated with PPF-CDNB and PPF-CDNB + H_2_S (Fig. 7a and d) showed no morphological changes in the cell structure, indicating the effectiveness of PPF-CDNB in detecting exogenous H_2_S.

The enzymes 3-mercaptopyruvate thiotransferase (3MST), cystathionine γ-lyase (CSE), and cystathionine β-synthase (CBS) present in the cytoplasm utilize Cys as a substrate to produce endogenous H_2_S.^48^ PPG is a potent H_2_S inhibitor of cystathionine γ-lyase (CSE) synthesis. For endogenous H_2_S detection, experiments were divided into three groups. In the first group, A549 cells were incubated with PPF-CDNB (10 μM) followed by the addition of cysteine (Cys) (50 μM), resulting in observable green fluorescence (Fig. 7h). As a control in the second group, A549 cells pretreated with PPF-CDNB (10 μM) were incubated with only 200 μM PPG, no fluorescence (Fig. 7k). In the third group, A549 cells were first incubated with PPF-CDNB (10 μM), followed by the addition of Cys (50 μM) and 200 μM PPG before imaging under light microscopy, confirming the absence of green fluorescence in the cytoplasm (Fig. 7n).

This highlights the role of PPG in inhibiting intracellular enzymes, thus preventing endogenous H_2_S production. In the absence of H_2_S in 2,4 dinitrobenzene ether will not cracking, PPF-CDNB remain inactive forms (no fluorescence). Bright-field images of A549 cells incubated with PPF-CDNB, PPF-CDNB + Cys, PPF-CDNB+ Cys + PPG, and PPF-CDNB + PPG showed no change in the morphology of the cellular structures (Fig. 7).

It should be noted that the weak fluorescence observed in PPF-CDNB treated A549 cells (Fig. 7b) may be due to the reaction of PPF-CDNB with a small amount of endogenous H_2_S present in the cytoplasm. Show that the PPF-CDNB endogenous H_2_S level is capable of detecting living cells.

## 4 Conclusions

We have established a novel molecular design strategy for fluorescent probes, which can target H_2_S and exhibit significant fluorescence enhancement. This design is based on asymmetric π-conjugated phosphine oxide, achieved through the creation of the large π-bond conjugated probe PPF-CDNB with a PPF structure. The diaryl ether structure on the synthesized PPF compound showed a blue shift of more than 100 nm when thiolated (Fig. S4a†), which could significantly reduce the interference of crosstalk signal and improve the accuracy of ratio measurement. Utilizing PPF-OH as a scaffold, we developed the PPF-CDNB fluorescence probe, which markedly amplified the fluorescence intensity of the H_2_S thiolysis reaction. Based on the complex environment *in vivo*, the selectivity experiment and anti-interference experiment of the probe were also conducted (Fig. 3b and c). These experiments revealed the probe's ability to distinguish H_2_S from other interfering substances, showcasing high selectivity and sensitivity. We believe that using this unique method of structural shift of PPF provides a flexible platform for the design of various fluorescent probes.

The rapid transformation of the probe in the green channel observed during live cell imaging indicates the cellular response of PPF-CDNB to H_2_S. Given its relevance to a myriad of biological phenomena and conditions, including Alzheimer's disease and down syndrome, PPF-CDNB is expected to serve as a valuable research tool for detecting related biological processes and diseases.

## Data availability

The data underlying this study are available in the published article and its ESI.†

## Author contributions

Shuntao Zhang: writing – manuscript, methodology, investigation, conceptualization. Xingyong Liu: resources. Xiangjun Chen: visualization. Jiefeng Tang: investigation. Juan Wang: writing – review editing, supervision, conceptualization.

## Conflicts of interest

There are no conflicts to declare.

## Supplementary Material

RA-014-D4RA02979H-s001

RA-014-D4RA02979H-s002

## References

[cit1] Long L., Cao S., Jin B., Yuan X., Han Y., Wang K. (2019). Food Anal. Methods.

[cit2] Park G., Jang M., Han M. S. (2023). RSC Adv..

[cit3] Tong X., Hao L., Song X., Wu S., Zhang N., Li Z., Chen S., Hou P. (2022). RSC Adv..

[cit4] Zhong K., Deng L., Zhao J., Yan X., Sun T., Li J., Tang L. (2018). RSC Adv..

[cit5] Okada T., Li H., Li Y., Gu B., Su W., Duan X., Xu H., Huang Z., Yao S. (2017). Anal. Methods.

[cit6] Chan Z., Bo Q., Zeng Y., Chen J., Yu T., Yi L. (2017). Chin. J. Org. Chem..

[cit7] Li H., Peng W., Feng W., Wang Y., Chen G., Wang S., Li S., Wang K., Zhang J. (2016). Chem. Commun..

[cit8] Kimura H. (2010). Amino Acids.

[cit9] Pak Y. L., Li J., Ko K. C., Kim G., Lee J. Y., Yoon J. (2016). Anal. Chem..

[cit10] Wang Q., Chen Z., Zhang X., Xin Y., Xia Y., Xun L., Liu H. (2021). Free Radical Biol. Med..

[cit11] Peng S., Zhong T., Guo T., Shu D., Meng D., Liu H., Guo D. (2018). New J. Chem..

[cit12] Long L., Yuan F., Yang X., Ruan P., Chen X., Li L., He D., Yang S., Yang Y., Wang K. (2022). Sens. Actuators, B.

[cit13] Kafle A., Bhattarai S., Miller J. M., Handy S. T. (2020). RSC Adv..

[cit14] Chen Z., Chen F., Sun Y., Liu H., He H., Zhang X., Wang S. (2017). RSC Adv..

[cit15] Yang M., Fan J., Du J., Peng X. (2020). Chem. Sci..

[cit16] Feng Q., Song Y., Ma Y., Deng Y., Xu P., Sheng K., Zhang Y., Li J., Wu S. (2023). Spectrochim. Acta, Part A.

[cit17] Chen W., Pacheco A., Takano Y., Day J. J., Hanaoka K., Xian M. (2016). Angew. Chem., Int. Ed..

[cit18] Xue D., Zhou R., Lin X., Duan X., Li Q., Wang T. (2019). RSC Adv..

[cit19] Kabil O., Banerjee R. (2014). Antioxid. Redox Signaling.

[cit20] Surya S. G., Bhanoth S., Majhi S. M., More Y. D., Teja V. M., Chappanda K. N. (2019). CrystEngComm.

[cit21] Jarosz A. P., Yep T., Mutus B. (2013). Anal. Chem..

[cit22] Doeller J. E., Isbell T. S., Benavides G., Koenitzer J., Patel H., Patel R. P., Lancaster J. R., Darley-Usmar V. M., Kraus D. W. (2005). Anal. Biochem..

[cit23] Wu J., Wu S.-X., Wu Y., Kan Y.-H., Geng Y., Su Z.-M. (2013). Phys. Chem. Chem. Phys..

[cit24] Zhu Z.-L., Chen W.-C., Ni S.-F., Yan J., Wang S. F., Fu L.-W., Tsai H.-Y., Chi Y., Lee C.-S. (2021). J. Mater. Chem. C.

[cit25] Combes-Chamalet C., Cristau H.-J., McPartlin M., Plénat F., Scowen I. J., Woodroffe T. M. (1997). J. Chem. Soc., Perkin Trans. 2.

[cit26] Nishimura K., Hirano K., Miura M. (2020). Org. Lett..

[cit27] Duffy M. P., Delaunay W., Bouit P.-A., Hissler M. (2016). Chem. Soc. Rev..

[cit28] Si E., Zhao P., Wang L., Duan Z., Mathey F. (2020). Eur. J. Org Chem..

[cit29] Kuninobu Y., Yoshida T., Takai K. (2011). J. Org. Chem..

[cit30] Onoda M., Koyanagi Y., Saito H., Bhanuchandra M., Matano Y., Yorimitsu H. (2017). Asian J. Org. Chem..

[cit31] Fujimoto H., Kusano M., Kodama T., Tobisu M. (2020). Org. Lett..

[cit32] Nishimura K., Hirano K., Miura M. (2019). Org. Lett..

[cit33] Cornforth J. (1996). J. Chem. Soc., Perkin Trans. 1.

[cit34] Nishimura K., Xu S., Nishii Y., Hirano K. (2023). Org. Lett..

[cit35] Sarac K., Orek C., Cetin A., Dastan T., Koparir P., Dastan S. D., Koparir M. (2016). Phosphorus, Sulfur Silicon Relat. Elem..

[cit36] Bonetti G., Arnaboldi S., Grecchi S., Appoloni G., Massolo E., Rossi S., Martinazzo R., Orsini F., Mussini P. R., Benincori T. (2020). Molecules.

[cit37] Dong Y., Takata Y., Yoshigoe Y., Sekine K., Kuninobu Y. (2019). Chem. Commun..

[cit38] Nishida J., Kawakami Y., Yamamoto S., Matsui Y., Ikeda H., Hirao Y., Kawase T. (2019). Eur. J. Org Chem..

[cit39] Hibner-Kulicka P., Joule J. A., Skalik J., Bałczewski P. (2017). RSC Adv..

[cit40] Chen R.-F., Zhu R., Fan Q.-L., Huang W. (2008). Org. Lett..

[cit41] Zhong D., Yu Y., Song D., Yang X., Zhang Y., Chen X., Zhou G., Wu Z. (2019). ACS Appl. Mater. Interfaces.

[cit42] Kim J. K., Bong S. Y., Park R., Park J., Jang D. O. (2022). Spectrochim. Acta, Part A.

[cit43] Fang B., Yang J., Wang L., Li H., Guo J., Zhang J., Guo Q., Peng B., Liu K., Xi M., Bai H., Fu L., Li L. (2024). Chin. Chem. Lett..

[cit44] Zhong K., He Y., Deng L., Yan X., Li X., Tang Y., Hou S., Tang L. (2020). Anal. Chim. Acta.

[cit45] Bhattacharyya A., Guchhait N. (2021). Chem. Phys..

[cit46] Pan Y., Li Y., Sun X., Tang L., Yan X. (2023). Dyes Pigm..

[cit47] Hoshino Y., Hanaoka K., Sakamoto K., Yasunaga M., Kojima T., Kotani D., Nomoto A., Sasaki E., Komatsu T., Ueno T., Takamaru H., Saito Y., Seto Y., Urano Y. (2022). RSC Chem. Biol..

[cit48] Kaur R., Kour R., Kaur S., Singh P. (2023). J. Photochem. Photobiol., A.

