# Supplementary figures and images for: A novel fluorescent probe with a phosphofluorene molecular structure for selective detection of hydrogen sulfide in living cells
Source: RSC Adv. 2024 Jul 2;14(29):20966–73. doi: 10.1039/d4ra02979h (PMC11218039; doi:10.1039/d4ra02979h)

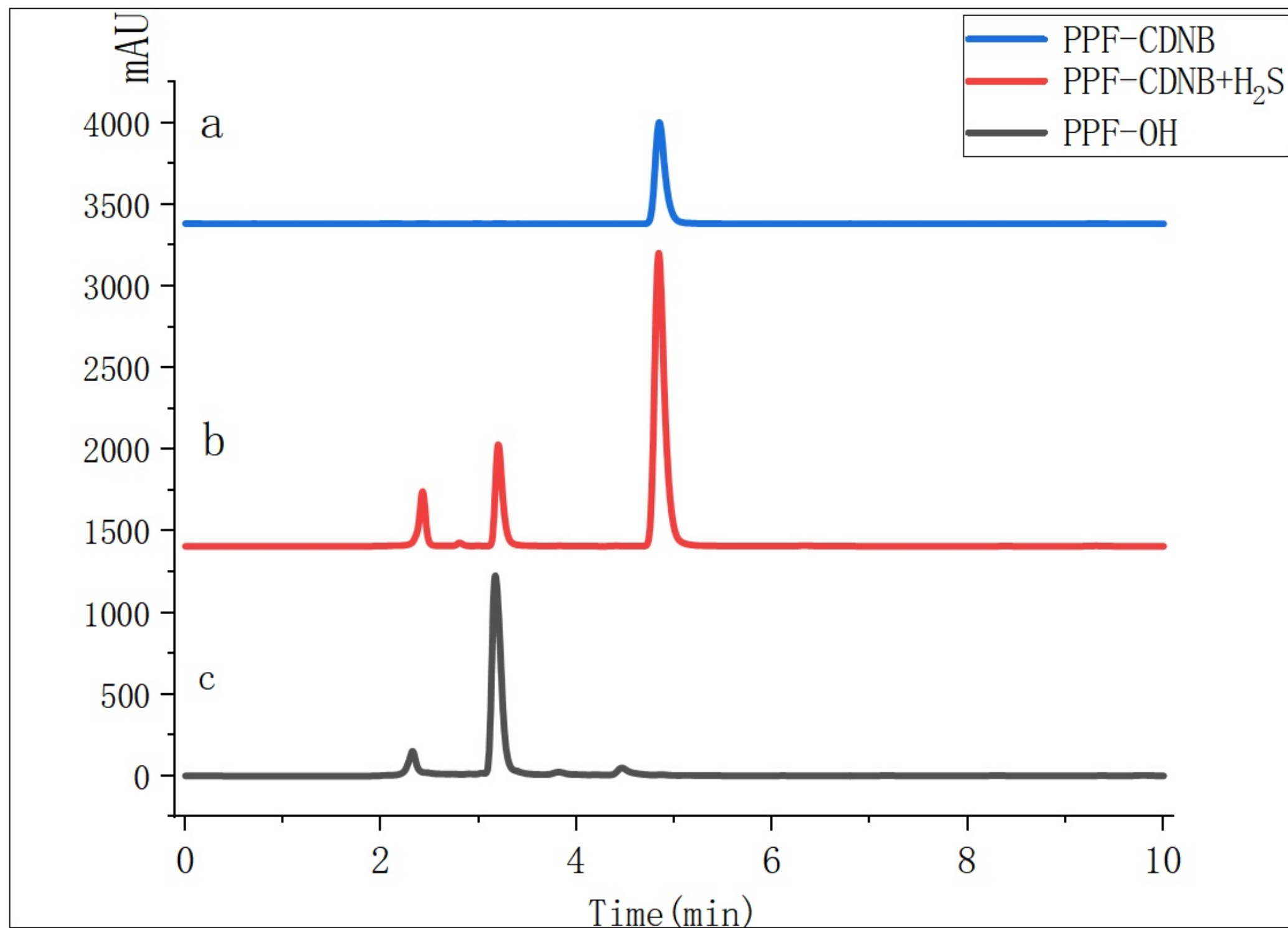

Supplement: RA-014-D4RA02979H-s001 [file RA-014-D4RA02979H-s001.pdf]
