# Supplementary material for: A novel fluorescent probe with a phosphofluorene molecular structure for selective detection of hydrogen sulfide in living cells
Source: RSC Adv. 2024 Jul 2;14(29):20966–73. doi: 10.1039/d4ra02979h (PMC11218039; doi:10.1039/d4ra02979h)
Supplement: RA-014-D4RA02979H-s002 [file RA-014-D4RA02979H-s002.pdf]

Electronic Supplementary Material (ESI) for RSC Advances.
This journal is © The Royal Society of Chemistry 2024

Electronic Supplementary Material (ESI) for RSC Advances.
This journal is © xxxxxxxx

Supporting Information

A novel fluorescent probe with a phosphofluorene molecular structure for selective detection of hydrogen sulfide in living cells

Shuntao Zhang, Xingyong Liu, Xiangjun Chen, Jiefeng Tang and Juan Wang*

College of Chemical Engineering, Sichuan University of Science & Engineering, No. 519, Huixing Road, High-tech Zone, Zigong 643000, China

*Corresponding Author: Juan Wang

Email: wangjuan2022@suse.edu.cn

Table of contents

Supporting Information 1
1. Synthesis process 2
2. Supplementary data 4
Figure S1 4
Figure S2: 4
Figure S3: 5
Figure S4 5
Figure S5： 6
Figure S6 6
Figure S7: 7
3. References 12

1. Synthesis process

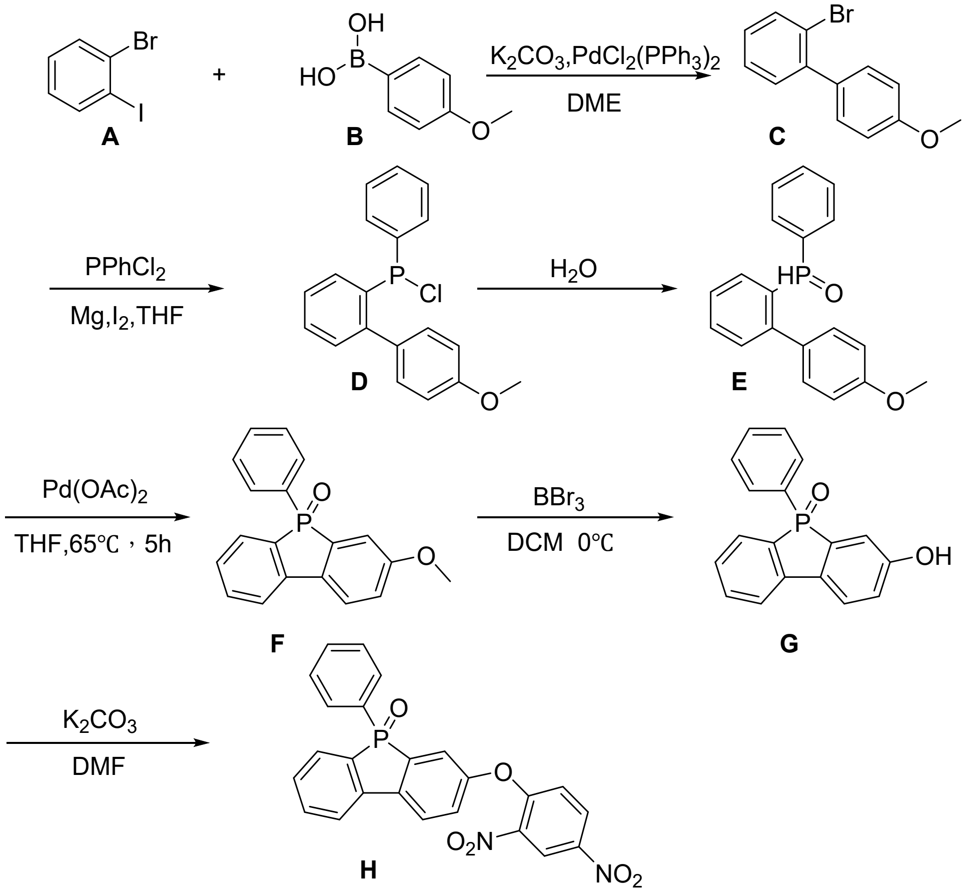

Preparation of compound **C** [1]

Compound A (5.66 g,20 mmol) and compound B (3.344 g,22 mmol) were mixed to obtain the mixture, Then K_2CO_3 (6.9 g,50 mmol) and $Pd(PPh)_3Cl_2$ (0.221 g,0.3 mmol) were added to the mixture, dissolved in 60 mL glycol dimethyl ether and 8 mL water, and then added to 250 mL round bottom flask. After reflux for 8 h at 85 °C under argon, TLC detection was performed. The transparent oil compound **C** (4.8 g,91.2 %) was obtained by extraction of ethyl acetate. ^{1}H NMR (600 MHz, $CDCl_3$) δ 7.58 (dd, J = 8.0, 0.8Hz, 1H), 7.30-7.22 (m, 4H), 7.09 (ddd, J = 8.0, 6.9, 2.2Hz, 1H), 6.90-6.87 (m, 2H), 3.78 (s, 3H).

Preparation of compound **E**[2,3]

Mg(515 mg,21.5 mmol) was added under argon, I_2 (163.83 mg, 0.645 mmol) was used as solvent with 20 mL THF, and stirred at 40 ℃ until iodine faded. Compound **C** (4.71 g,17.9 mmol) was dissolved in 10mL of THF and slowly added to a three-necked flask (at least 5min), heated to 80 ℃ for 2h, and cooled to room temperature. $PPhCl_2$ (4.8 g,26.85 mmol) dissolved in 10 mL THF solution was slowly added to a

clean round-bottom flask under argon gas condition (at least 5 min), heated to 80 ℃ and refluted for 3 h, detected by TLC, cooled to 0 ℃, oxidized with H_2O, and stirred for 15 min. Et_3N (10 mL) was added and stirred for 20 min, then detected by TLC and extracted with ethyl acetate to obtain compound E (2.6 g,47.1 %). ^1H NMR (600 MHz, $CDCl_3$) δ8.27 (s, 1H), 7.89 (dd, J = 14.1, 7.6 Hz, 1H), 7.56 (t, 1H), 7.47 (d, J = 7.6 Hz, 1H), 7.45 (s, 1H), 7.41 (t, 1H), 7.36 7.27 (m, 5H), 7.16 (d, J = 8.6 Hz, 2H), 6.82 (d, J = 8.7 Hz, 2H), 3.81 (s, 3H).

Preparation of compound **F**[4,5]

Compound **E** (1.019 g,3.3 mmol) was dissolved in 20 mL THF under argon and added to a round-bottom bottle. $Pd(OAc)_2$ (37 mg,0.165 mmol) was added as catalyst. The mixture was stirred at 65 ℃ for 5h. ^1H NMR (600 MHz, $CDCl_3$) δ 7.73-7.68 (m, 2H), 7.68-7.62 (m, 3H), 7.53 (t, 1H), 7.48 (td, 1H), 7.38 (td, J = 9.2 Hz, 2H), 7.29 (td, 1H), 7.20 (dd, J = 10.9 Hz, 1H), 7.08 (dd, J = 8.5 Hz, 1H), 3.79 (s, 3H).

Preparation of Compound **G**

Compound **F** (1.12 g,3.6 mmol) was dissolved in 20 mL dichloromethane under argon and added to a round-bottom flask. BBr_3 (1.09 g,4.35 mmol) was slowly added as catalyst at 0 ℃ and stirred for 4 h at 0 ℃. The compound G (746 mg,71 %) was extracted as yellow oil. ^1H NMR (600 MHz, $CDCl_3$) δ 10.18 (s, 1H), 7.97-7.89 (m, 2H), 7.63 (p, J = 26.5 Hz, 2H), 7.58-7.49 (m, 3H), 7.47 (td, H) 2H), 7.34 (td, J = 9.1Hz, 1H), 7.04 (ddd, J = 24.2 Hz, 2H).

Preparation of compound **H** [6-8]

Compound **G** (200 mg,0.68 mmol) and 2,4-dinitrophenyl chloride (166.4 mg,0.82 mmol) were dissolved in 7 mL DMF and added to a round-bottom flask. K_2CO_3 (141 mg,1.03 mmol) was added and stirred at 100 ℃ for 6 h. A yellow solid compound H (267 mg,85.6 %) was obtained, which was 3-(2,4-dinitrophenoxyl) -5-phenylbenzo [b] fosinarido-5-oxide, phospho fluorene. ^1H NMR (600 MHz, $CDCl_3$) δ 8.78 (d, J = 2.7 Hz, 1H), 8.28 (dd, J = 9.2, 2.7 Hz, 1H), 7.86 (dd, J = 8.4, 3.2 Hz, 1H), 7.78 (dd, J = 7.7, 2.8 Hz, 1H), 7.68 (dd, J = 9.8, 7.6 Hz, 1H), 7.57 (dt, J = 8.2, 4.4 Hz, 4H), 7.47 (td, J = 7.4, 1.2 Hz, 1H), 7.41 7.34 (m, 5H), 7.29 (dd, J = 8.4, 2.2 Hz, 1H), 7.06 (d, J = 9.2 Hz, 1H).^{13}C NMR (150 MHz, $CDCl_3$) delta 154.04 , 153.69 , 153.59 , 141.05 , 139.68 , 139.55 , 138.81 , 138.67 , 132.88 , 131.74 , 130.04 , 129.96 , 129.28 , 129.22 , 128.85 , 128.78 , 128.05 , 128.03 , 127.94 , 124.30 , 122.44 (d, J = 11.5 Hz), HRMS(ESI): $C_{24}H_{15}N_2O_6NaP$ for $[M+Na]^+$, calculated 481.0565, found 481.0570.

2. Supplementary data

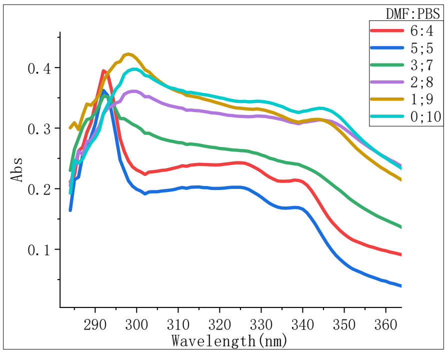

Figure S1: UV absorption spectra of 20 μM **PPF-CDNB** fluorescent probe in PBS buffer solution supplemented with DMF at different concentrations

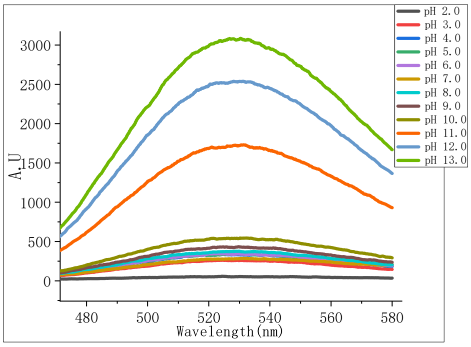

a

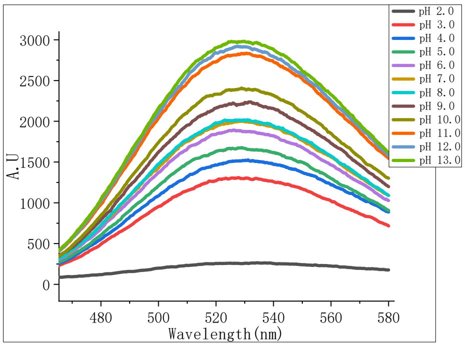

b

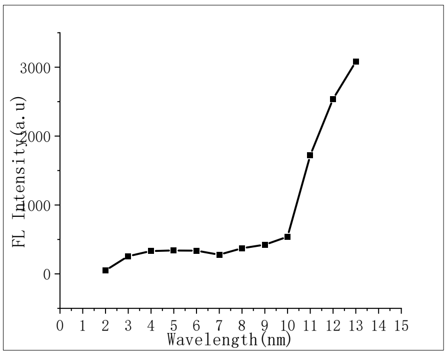

c

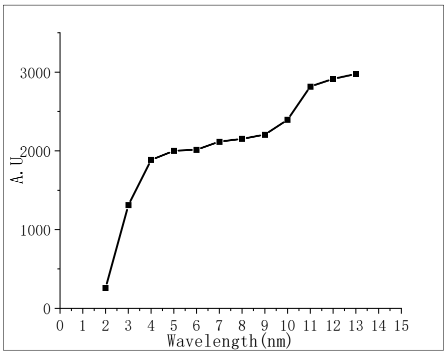

d

Figure S2: fluorescence intensity of 20μM probe **PPF-CDNB** at different pH values in a buffer solution system of PBS/DMF (V/V=9/1, pH7.4); the pH was adjusted with NaOH and HCl and set to 1.0 for normalization. (a) Fluorescence intensity of probe **PPF-CDNB** at different pH values (pH 2-13). (b) Fluorescence intensity of probe **PPF-CDNB**+H_2S at different pH values (pH 2-13). (c) Gradient curve of fluorescence intensity of probe **PPF-CDNB** at 528 nm at different pH values (pH 2-13). (d) Gradient curve of fluorescence intensity of probe **PPF-CDNB** + H2S at 528 nm at different pH values (pH 2-13).

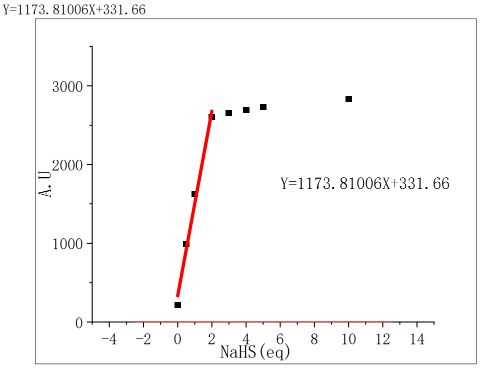

309.854	321.568	326.516	337.84
312.496	321.731	345.182	328.551

Standard deviationσ=11.91371549

Slope of slopek =1173.81006

LOD =3σ/k

The detection limit of the fluorescent probe was 150 nM

Figure S3: Detection limits of fluorescent probes

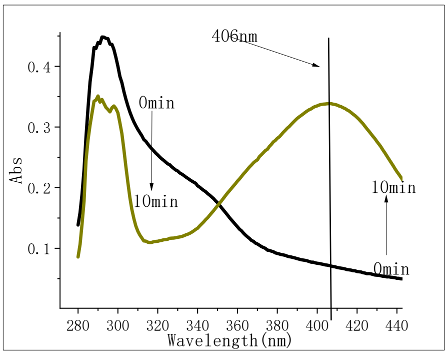

a.

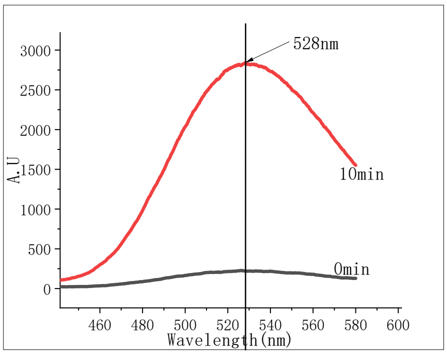

b.

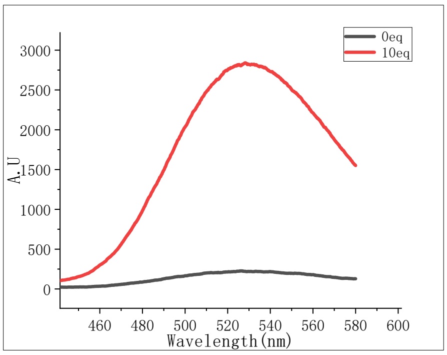

c.

Figure S4: (a) UV absorption spectra of 20 μm probe **PPF-CDNB** at 0 min and 10 min after 10 eq NaHS were recorded. (b) The fluorescence emission spectra of 20 μM probe **PPF-CDNB** were measured at 0 min and 10 min after the addition of 10 eq NaHS. (c) Fluorescence emission spectra obtained 15 min after 0eq and 10eq NaHS were added to 20 μM **PPF-CDNB**. The excitation wavelength was set at 293 nm in PBS/DMF (V/V=9/1, pH7.4) buffer solution.

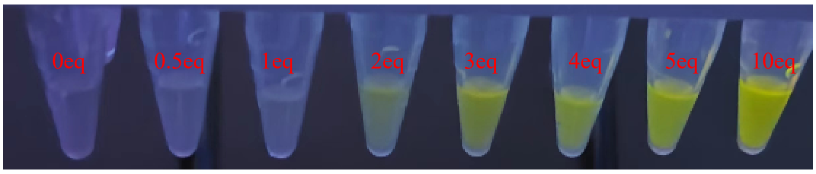

Figure S5：The UV map of the 20 μM probe **PPF-CDNB** was obtained after adding NaHS concentration (0-10 eq) for 15 minutes in a buffer solution of PBS/DMF (V/V=9/1, pH7.4), and observed under UV lamp conditions at 365 nm.

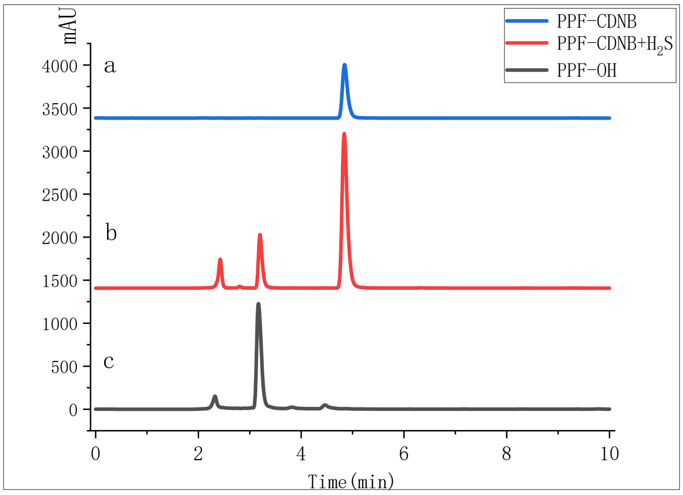

Probe **PPF-CDNB** the peak position in the HPLC is 4.789 min, under the condition of the same measure of PPF-OH the peak position is 3.141 min, to probe **PPF-CDNB** join after NaHS 0.5 eq, the location of the probe in 4.789 min and 3.141 min respectively near the peak, Moreover, a single peak also appears at 2.413 min, which is 2,4-dinitrobenzenethiol formed by partial cleavage of **PPF-CDNB** under the action of H_2S, It's a powerful evidence (Fig. 6I).

Figure S6 Results of HPLC titration experiments for probe **PPF-CDNB.** (a) **PPF-CDNB**, (b) **PPF-CDNB**+NaHS, (c) **PPF-OH**. The mobile phase consisted of CH_3CN/H_2O with a gradient elution as follows: 0-10 min, 70/30. The flow rate was set at 0.8 mL/min and the temperature at 30°C. Detection was performed at a wavelength of 254nm with an injection volume of 5.0 μL.

CCK-8 was used to detect the toxicity

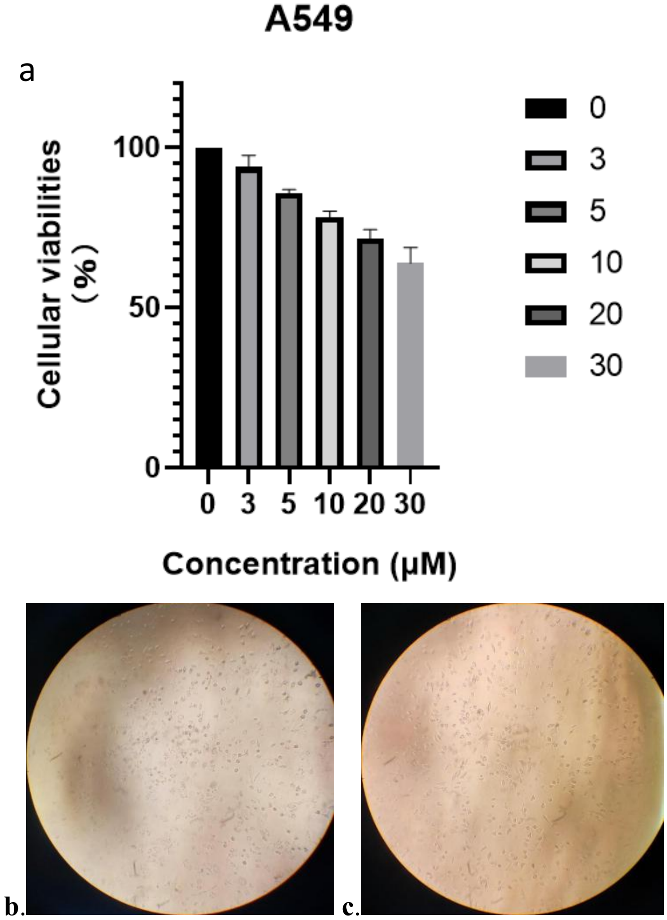

(b) Before addition of probe **PPF-CDNB.** (c) After addition of probe **PPF-CDNB** for 12 h in culture

Figure S7: The cytotoxicity of probe **PPF-CDNB** was investigated using A549 cells (lung tumor cells) and CCK-8 reagent. A549 cells (lung tumor cells) were seeded and incubated in 96-well plates for 24 h (37 °C, 5% CO_2) before detection. After removing the old medium, the same volume and different concentrations of probe **PPF-CDNB** (0 μM, 3 μM, 5 μM, 10 μM, 20 μM, 30 μM) were added and incubated for 12 hours according to the above conditions. The original medium was aspirated and then washed gently three times with PBS buffer. Next, CCK solution (0.500 mg/mL, 100 μL) was added to each well. After 4h of culture, cytotoxicity was calculated by measuring the absorbance at 450 nm using a microplate reader.

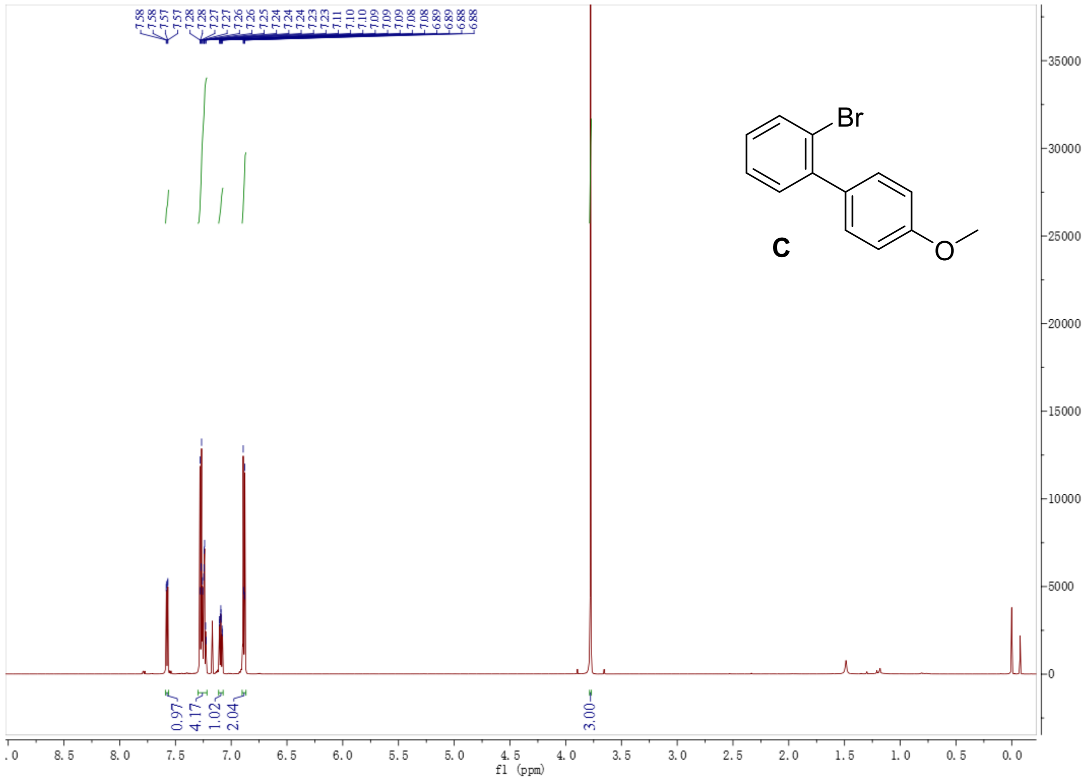

^{1}H NMR spectrum (600 MHz, $CDCl_3$) of C.

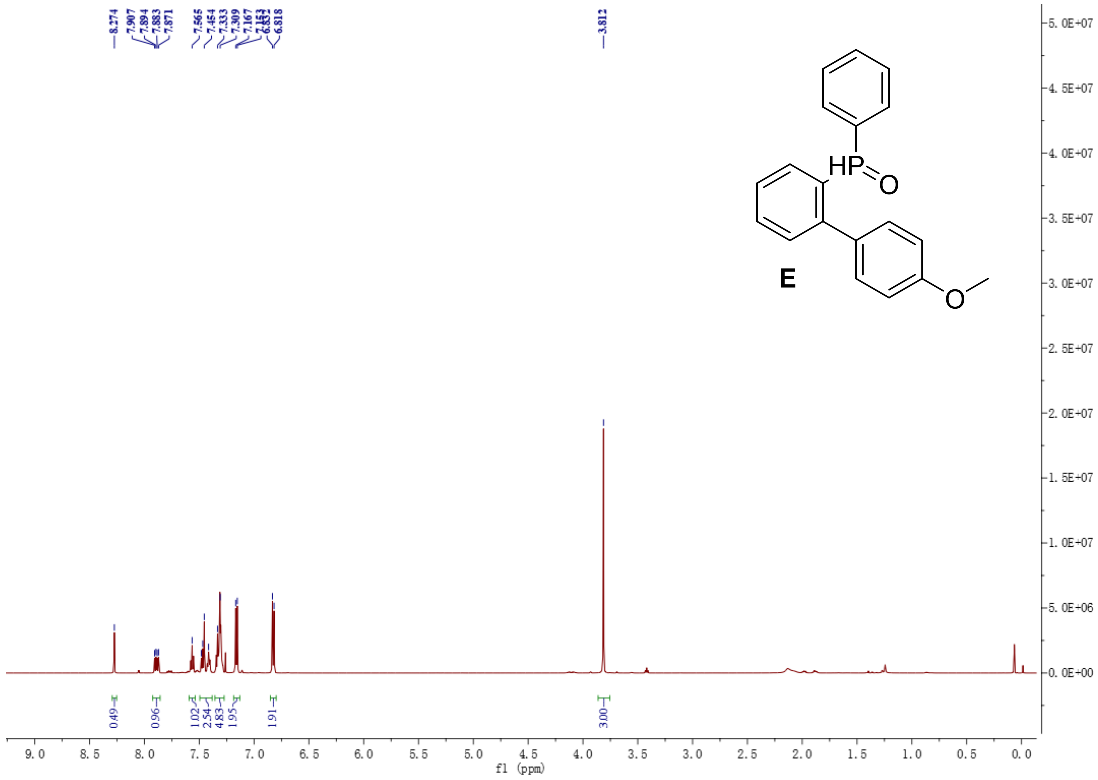

^{1}H NMR spectrum (600 MHz, $CDCl_3$) of **E**.

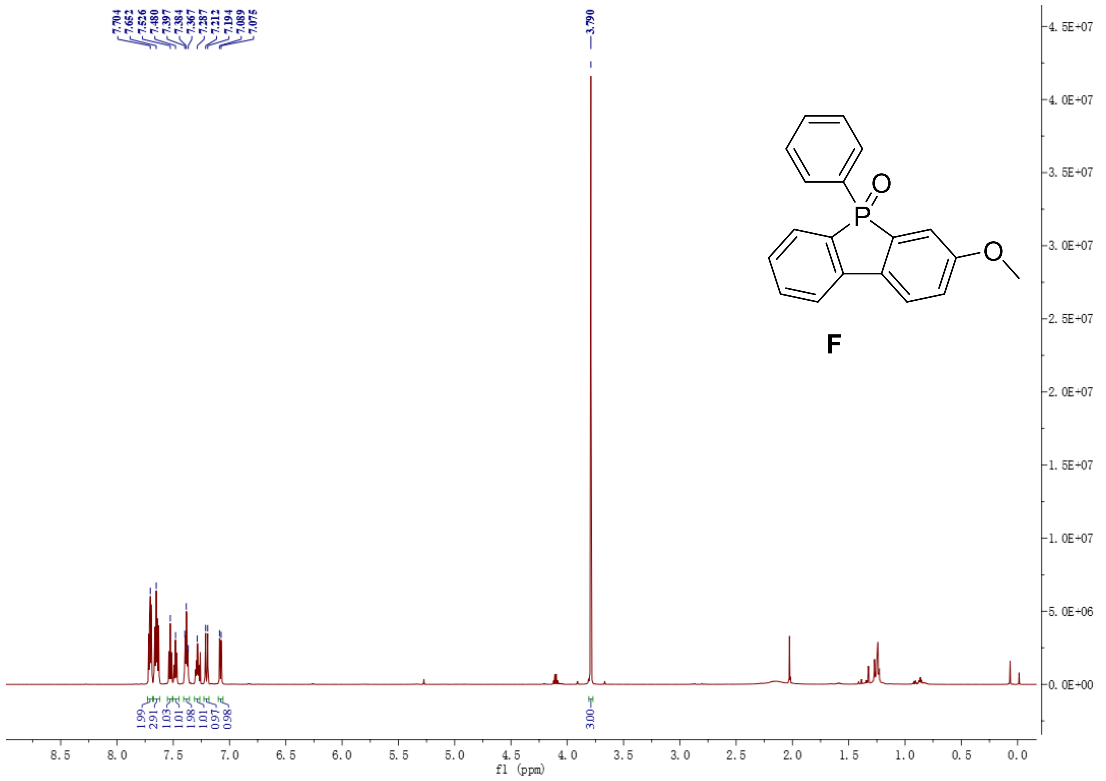

^{1}H NMR spectrum (600 MHz, DMSO) of **F**.

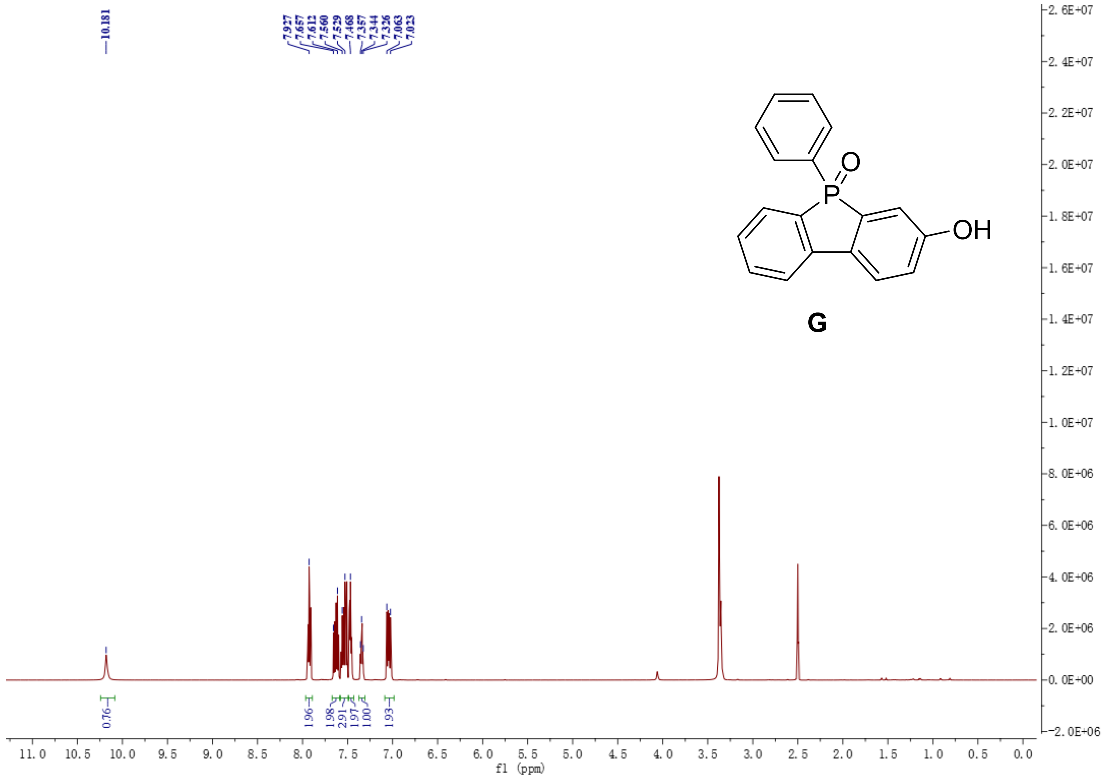

^{1}H NMR spectrum (600 MHz, DMSO) of **G**.

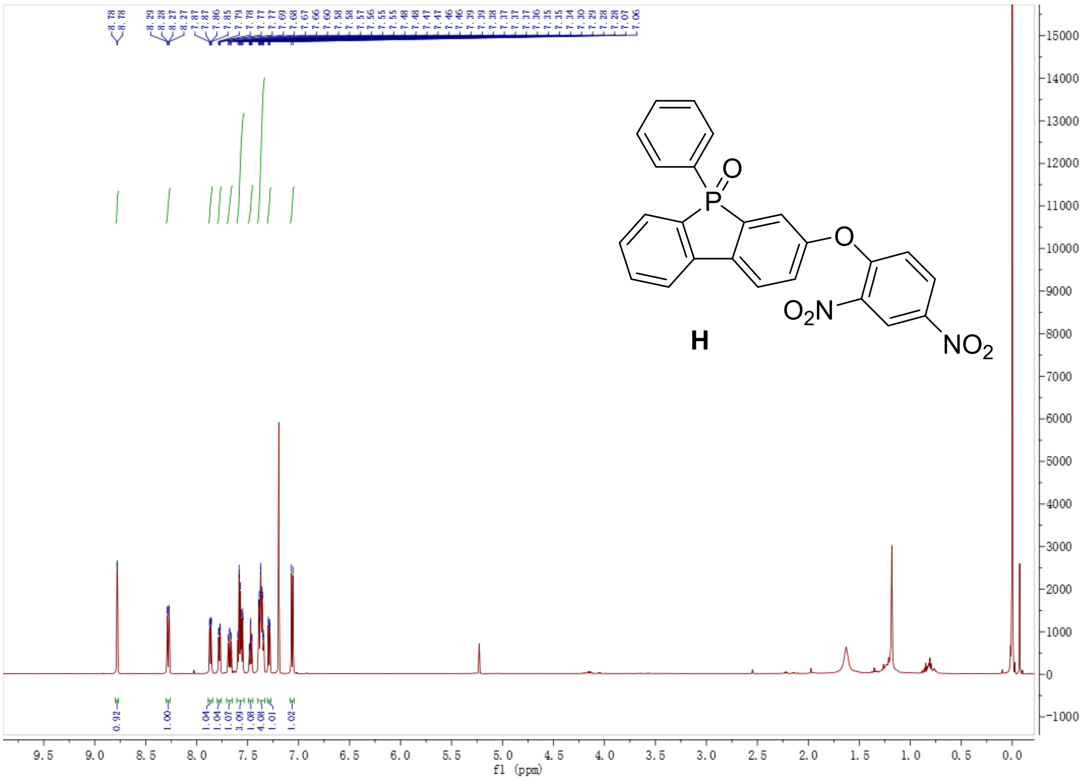

^{1}H NMR spectrum (600 MHz, $CDCl_3$) of **H**.

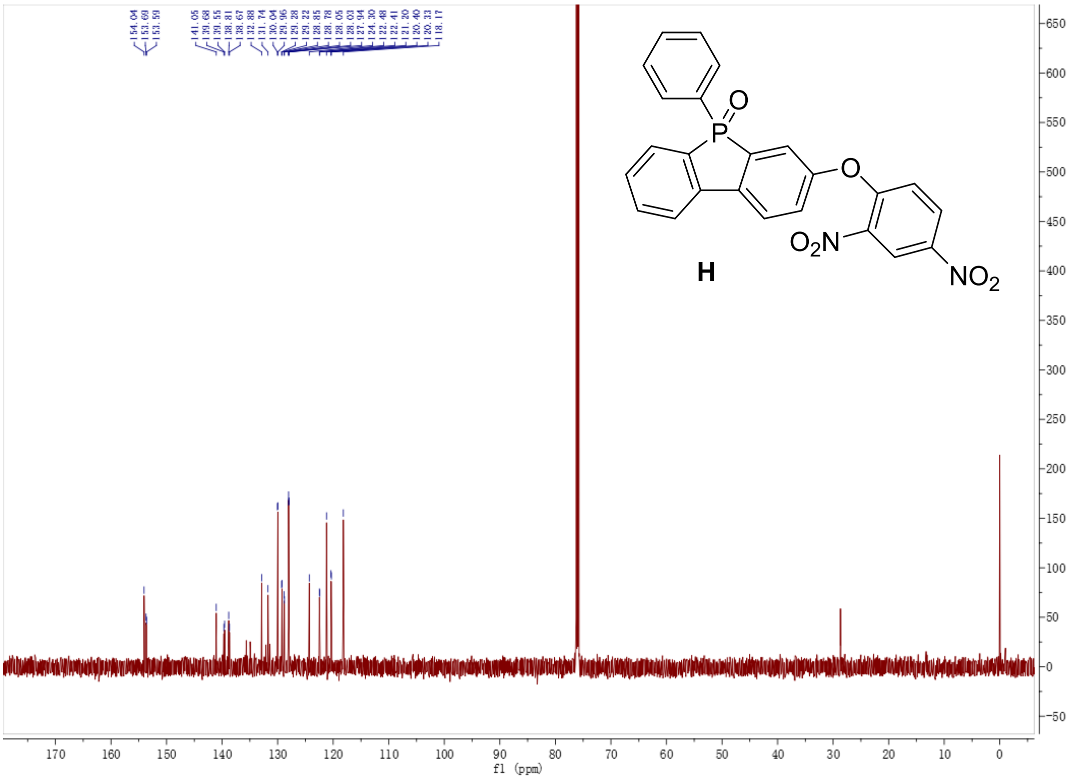

^{13}C NMR spectrum (150 MHz, $CDCl_3$) of **H**

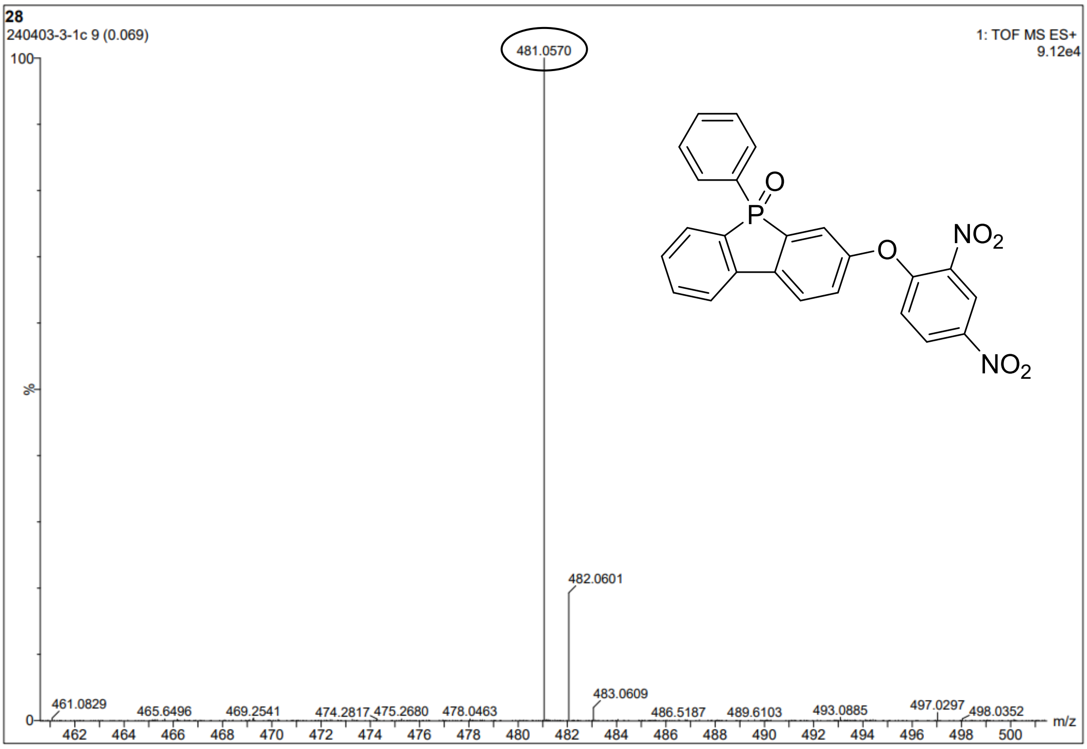

Single Mass Analysis
Tolerance = 5.0 mDa / DBE: min = -1.5, max = 50.0
Element prediction: Off
Number of isotope peaks used for i-FIT = 3

Monoisotopic Mass, Even Electron Ions
1558 formula(e) evaluated with 1 results within limits (up to 50 best isotopic matches for each mass)
Elements Used:
C: 24-24 H: 15-15 N: 0-100 O: 0-100 Na: 0-1 P: 1-2

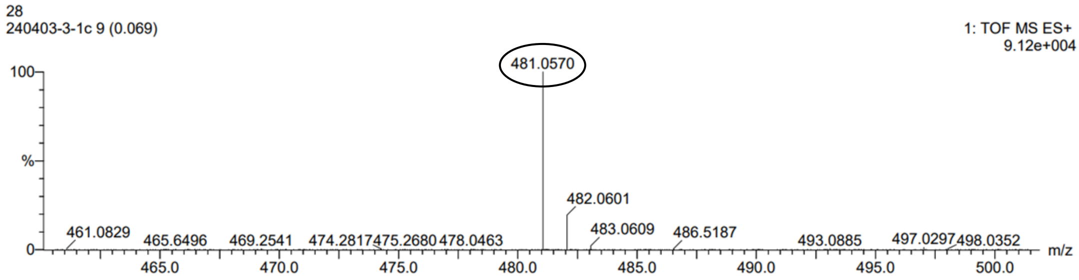

Minimum:				-1.5				
Maximum:		5.0	10.0	50.0				
Mass	Calc. Mass	mDa	PPM	DBE	i-FIT	Norm	Conf(%)	Formula
481.0570	481.0565	0.5	1.0	18.5	269.3	n/a	n/a	C24 H15 N2 O6 Na P

Mass spectrum of probe **PPF-CDNB**

3. References

S1 G. Meng and M. Szostak, *Org. Biomol. Chem.*, 2016, **14**, 5690–5707.

S2 M. Onoda, Y. Koyanagi, H. Saito, M. Bhanuchandra, Y. Matano and H. Yorimitsu, *Asian J Org Chem*, 2017, **6**, 257–261.

S3 H. Fujimoto, M. Kusano, T. Kodama and M. Tobisu, *Org. Lett.*, 2020, **22**, 2293–2297.

S4 E. Si, P. Zhao, L. Wang, Z. Duan and F. Mathey, Eur. *J. Org. Chem.*, 2020, **2020**, 697–701.

S5 K. Nishimura, K. Hirano and M. Miura, *Org. Lett.*, 2020, **22**, 3185–3189.

S6 F. M. Moghaddam and M. Eslami, *Appl Organometal Chem.* 2018;**32**:e4463..

S7 H. Fu, X. Liu, Y. Jiang and Y. Zhao, *Synlett.*, 2008, **2008**, 221–224.

S8 S. G. Babu and R. Karvembu, *Tetrahedron Lett.*, 2013, **54**, 1677-1680.
